# Keyword Index for Volume 101

**DOI:** 10.1038/sj.bjc.6605476

**Published:** 2009-12-08

3D culture 658

4E-BP1 424

5-FU 1846

6-thioguanine 269

7q34 722

A20 1555

*α*-catenin 1574

acute myeloid leukaemia 363, 743

ADAM17/TACE 457

ADCC 1758, 1807

ADCP 1758

adenocarcinoma 1, 492, 855

adenoma 1124

adjuvant chemotherapy 908, 1282

Adjuvant! Online 1074

adjuvant therapy 875, 1074

adolescent girls 1502

advanced cancer 759

advanced gastric cancer 1261

advanced pancreatic cancer 1162

aetiology of breast cancer 1213

Africa 202

age at first pregnancy 865, 1213

age at first sexual intercourse 865

aggressive disease 522, 1891

AIB1 1253

Akt 342

AKT1 145

ALCAM 457

alcohol 1630

alkylating agent 441

alpha-blockers 843

amplification 973

amplification refractory mutation system 1724

anaemia 1961

androgen signalling 1949

androgen withdrawal 1731

androgen-independent progression 1731

androgens 498

angiogenesis 64, 278, 605, 666, 957, 1749, 1884, 1986

Annexin II 287, 1145

anthracyclines 792, 1549

anti-angiogenesis 418, 1183, 1233

antibody 71, 1709, 1807

AP-2α 637

apoptosis 342, 410, 628, 684, 765, 774, 792, 1290, 1307, 1410, 1585, 1606, 2005

APRIL 1051

aromatase 1613

aromatase inhibitors 875, 1253

ASCUS 511

asymptomatic 1522

atacicept 1051

ATL 1497

ATM/ATR 357, 441

attenuated Salmonella typhimurium 1683

attitude 250

Aurora A 504

autophagy 1402, 1585

axitinib 1162

AZD6244 1724

*β*-catenin 1596

B7-H3 1709

Barrett's oesophagus 1

basement membrane 91

Bax 116

Bcl-3 1555

benign prostatic hyperplasia 843

benign tumours 303

benzoporphyrin-derivative monoacid ring A (BPD-MA) 2015

bevacizumab 238, 1033, 1986

BF_2_-chelated tetraaryl-azadipyrromethene 1565

bile duct cancer 1846

biliary tract 621

bioavailability 1290

biodistribution 1565

biomarkers 1, 91, 295, 335, 410, 1107, 1162, 1175, 1194, 1338, 1676, 1957

birth interval 1213

bladder cancer 98, 1091, 1316

BLyS 1051

BMI 534

bone malignant fibrous histiocytoma 1425

bone metastasis 263

bone tumour 1909

brachytherapy 1839

BRAF 465, 715, 722, 1448, 1724

brain metastases 1919

BRCA1 32, 1168, 1651, 2048

BRCA1/2 1456

BRCA1-associated tumours 1469

BRCA2 32, 2048

breast cancer 38, 48, 160, 166, 178, 278, 403, 582, 589, 598, 605, 666, 673, 875, 902, 1005, 1074, 1168, 1253, 1307, 1329, 1338, 1345, 1351, 1456, 1522, 1529, 1606, 1613, 1651, 1692, 1769, 1900, 1919, 1925, 2048

breast cancer chemotherapy 1978

breast neoplasm 395

breast screening 1338

breastfeeding 860

BRIP1 2043

BubR1 504

c Kit 48

C3H/He mice 363

CA 19-9 1162

CA IX 645

cAMP 2038

cancer cell lines 256

cancer dynamics 1130

cancer ecology 1130

cancer incidence 1202

cancer registry 215, 1925

cancer stem cell 320, 2030

cancer stem-like cell 1425

cancer survival 561, 568, 1939

cancer-initiating cell 1425

cannabinoids 940

cannabis 1207

cap-dependent 424

capecitabine 12, 924, 1039

carbonic anhydrase 645

cardiac valves 575

cardiomyopathy 575

cardioprotection 792

cardiotoxicity 792

case–control study 185, 1316, 1641

caspases 342, 441

cathepsin D 1175

CB_2_ receptor 940

CD166 457

CD26 983

CD44 432

CD45 983

CD49b 320

CD81 1402

CD82 1402

CD8+ lymphocytes 1382

cDNA microarray 1365

CEA 1758

cell cycle 1, 628, 637, 957, 1461

cellular senescence 1900

ceramide 940

cervical cancer 174, 202, 782, 816, 865, 967, 1059, 1635

cervical high-grade squamous intraepithelial lesion 27

cervix 871

cetuximab 715, 782, 1261

chemoprevention 829

chemoradiation 116, 782

chemoradiotherapy 1853

chemotherapy 225, 410, 621, 998, 1059, 1155, 1549

chemotherapy-induced neutropenia 1537

childhood cancer 55

China 1635

cholangiocarcinoma 621

chromatin decondensation 1044

chronic bacterial inflammation 1740

chronic lymphatic leukaemia 1444

circulating free DNA 1724

circulating tumour cells 589, 1813

circulation 91

cisplatin 225, 269, 621, 1059, 1261, 1853

CK-19 589

c-KIT 1995

CKS 1085

class III *β*-tubulin 951

clear-cell carcinoma 287

clinical audit 395

clinical trial 1044, 1978

clonality 1513

CMF 598

coagulation factor VII 2023

cohort study 1199, 1630, 1641, 1932

collagen 320, 492

colon cancer 1124, 1596, 1749

colonoscopy 1274

colorectal adenoma to carcinoma progression 707

colorectal cancer 12, 106, 250, 320, 473, 483, 557, 699, 715, 765, 822, 849, 916, 992, 998, 1033, 1039, 1269, 1274, 1282, 1374, 1382, 1664, 1671, 1758

colospheres 473

combination therapy 598

combined modality therapy 1039

comorbidity networks 749

comparative effectiveness research 882

computerised tomography 882

congenital abnormalities 518

consent procedure 1505

contrast agent 1884

cost-effectiveness 12, 882

COX-2 1448

Coxsackie and adenovirus receptor 1574

CRC 1671

CT scan 1066, 1522

curative surgery 557

curative-intent 1033

curcumin 1585

cutaneous melanoma 1724

CVD 403

CXC-chemokines 1620

CXCL8 1620

cycling 1932

cyclooxygenase-2 106, 483

cyclosporin 1307

CYGB 139

CYP2C8 1817

CYP2C9 1817

cytokeratin8 (CK8) 1298

cytokeratin18 (CK18) 410, 1298

cytokine 1019

cytology 871

cytoreduction 1066

DAP3 132

dasatinib 38, 263, 1699

data collection 395

DCIS 1925

death certificate 1085

dedifferentiation 1782

delta-like ligand 4 1749

deprivation 198

detection 992

dexrazoxane 792

DHA supplementation 1978

diagnosis and prognosis marker 98

diagnostic imaging 1225

Dicer 673

diet and nutrition 916

diffuse-type gastric carcinoma 1100

disease management 390

disease progression 951

disease-free survival 1817

disseminated tumour cells 589

distance 897

DNA copy number changes 707

DNA damage 452, 1290

DNA ploidy 1011

DNA topoisomerases, type II 1044

DNA-interfering drugs 350

DNA-PK 816

DNMT3A 699

docetaxel 1248, 1261

dose-dense 1549

dose-limiting toxicity 1241

Down syndrome 860

downregulation 1891

doxorubicin 219, 1044

drug efficacy 1565

drug resistance 1824

drug sensitivity 350

DTL/RAMP 691

dyskerin 1410

E1A 1555

EBV 1019

education 865

efficacy 357

eIF4E 424

eIF4F 424

elective 557

embryonal carcinoma 64

EMT 1769

endocrine therapy 1529

endometrial cancer 145, 335, 534, 537

endometrioid adenocarcinoma 534

endoscopy 1, 1580

endothelial 957

Epac 2038

Eph2A 1699

epidemiology 178, 534, 843, 916, 1085, 1199, 1202, 1274, 1630, 1635

epidermal growth factor receptor 149, 1114, 1949

epithelial cells 1596

epithelial ovarian cancer 432

epithelial–mesenchymal transition 320, 1365

epoetin-*β* 1961

epothilone 1241

Epstein–Barr virus 526, 530

ERCC4 2048

ERCC5 357

ERK 312

ER-stress 1402

ES cell transplantation 64

ethnic minority 250

evolutionary game theory of cancer 1130

Ewing sarcoma 55, 80

EWS–FLI1 80

ex vivo model 473

expression 973, 1351

extracellular matrix 91

EZH2 1282

faecal occult blood test 1274

FAK 327, 1699

familial risk 935, 1792

family history 1163, 1792

fascin 1124

fatality 198

Fc receptor 1807

fibroblast growth factor 1891

fibroblast growth factor receptor 3 (FGFR3) 2030

fibroblasts 106

FIH 1168

finasteride 843

first-line chemotherapy 232

fish consumption 849

flow cytometry 1011

fluorescence diagnosis 1580

FOLFOX 12

follow-up 561, 568

Foscan 658

founder mutation 32

FOXG1 1433

frizzled-7 1374

fucose 1807

fumes 1207

functional screen 1824

gain of function 1606

gallbladder cancer 621, 1846

gap junctions 829

gastric cancer 295, 530, 1011

gastric fibroblast 1365

gastrointestinal adenocarcinomas 410

gastrointestinal stromal tumour 7

GATA 1481

gemcitabine 621, 628, 637, 908, 1658, 1846, 1853

gene expression 498, 1393

gene expression profile 1469, 1824

genetic screening 32

genetic variation 2043

genome-wide expression 1782

genotyping 511

germ cell tumours 518

germ-line mutation 32

GIST 7

GJIC 829

glioblastoma 124, 615, 1986, 1995

gliomas 973, 124

glucocorticoids 1316

glycoengineering 1758

glycogen synthase kinase-3*β* 2005

gold(III) porphyrin 1a 342

G-proteins 498

granulocyte colony-stimulating factor 1549

growth hormone 303

Guinea 202

HBZ 1497

head and neck 418

head and neck squamous cell carcinoma 139

heat shock protein 27 1137

height 522

HER2 149, 232, 1253, 1357, 1676

hereditary breast-ovarian cancer 32

HIF-1*α* 139, 666, 1168

HIFU 19

high-intensity-focused ultrasound 19

histological grade 1925

histone deacetylases 1044

histone methylation 1798

histopathology 897

HLA class I 1321

Hodgkin's lymphoma 582, 1393

hormone receptor status change 1529

hormone therapy 951

HPV E6 oncoprotein 1351

HSP47 492

Hsp90 inhibitors 1620

HTLV-1 1497

human kallikrein-related peptidase 13 1107

human papillomavirus (HPV) 27, 202, 511, 865, 871, 1345, 1351, 1502, 1635, 1798

hypericin 1580

hyperplasia 666

hypoxia 139, 645, 1290, 1749, 1769, 2023

IBD 1671

idiopathic venous thromboembolism 840

ifosfamide 1059

IGF-1/IGF-1R 80

IGF-I 424

IL-4 1114

image analysis 1580

image cytometry 1011

imaging 2015

imatinib mesylate 1995

immune escape 1497

immunoediting 381

immunohistochemistry (IHC) 166, 967, 1282, 1298, 1338

immunoprecipitation 1145

immunosuppression 1444

immunosuppressive therapy 1316

immunosurveillance 381

immunotherapy 1709

immunotoxin 1114, 1307

indirect comparison 238

infections 860

inflammation 1

inflammatory bowel disease 1671

innate immune system 1329

insertion mutagenesis 1824

insulin-like growth factor 278

insulin-like growth factor-binding protein-4 278

integrin 320

integrin-*α*4 1365

interaction 1574

interferon 615

interleukin-2 1869

interleukin-6 1731

interval debulking surgery 244

interval from birth to cancer 1213

invadopodia 38

invasion 38, 983

irinotecan 924, 1039, 1658, 1972

iron 178

Japan 849

JFCR-45 panel 350

JPA 722

Ki-67 116, 166

ki-76 1282

kidney cancer 287

kinase inhibitors 829, 1717

koilocytosis 1345, 1351

Korea 526

KRAS 465, 715

L2DTL 691

landmark analysis 1537

lapatinib 1676

late-radiation damage 403

lenalidomide 803

leukaemia 860

leukotriene D4 1596

LGA 722

lifestyle 537

liposome 1884

liquid-based cytology (LBC) 511

liver metastasis 822

LSIL 511

lung cancer 327, 882, 897, 1919

lymph node metastasis 1100

lymphangiogenesis 605

lymphocytes 1513

lymphoma 575, 582

macrophages 106

Mage-b DNA vaccine 1329

magnetic resonance spectroscopy 1860

malignant glioma 1986

malignant pleural mesothelioma 1869

management 215

MAP kinase 983

mass screening 395

mathematical model 363

matrix metalloproteinase 1365

maximum tolerated dose 1241

MCF-7 cells 658, 1613

MDM2 774, 1456

MDM2 polymorphism 350

meat 178

melanoma 387, 551, 1448

melatonin 1613

Merkel-cell carcinoma 1444

Merkel-cell polyomavirus 1444

mesenchymal stem cells 1909

mesothelioma 1114

meta-analysis 149, 1641

metastasis 7, 287, 327, 432, 473, 749, 803, 998, 1033, 1307, 1329, 1374, 1664, 1949

metastatic breast cancer 232, 1676

metastatic colorectal cancer 465, 1972

methylation 124, 139

methylcholanthrene 381

metronomic chemotherapy 1986

MGMT 124

microarray 551, 1357

microenvironment 1393

micrometastasis 589

microRNAs 743

migration 1124

minimal residual carcinoma 418

miRNA 143, 551, 699, 743

miRNA-17-92 cluster expression 707

mitochondrial activity 1596

mitochondrion 132

mitotic assembly deficient protein 2 (MAD2) 1900

mitotic catastrophe 1585

MMP-9 983, 1876

MMR 269

models of care 561

molecular alterations 743

molecular imaging 1565

molecular signatures 1782

molecular switch 1949

molecular therapeutics 1114

monocyte-derived macrophages 1758

monotherapy 1676

mortality 174, 1085

mouse models 1651

MRI 924, 1884

mRNA 673

mtDNA transcription 1596

MUC4 1155

mucin 637

multiethnic population 185

multiforme 615

multiple drug-resistance proteins 432

multiple myeloma 1051, 1130, 1402

multiple-causes-of-death 1085

mutations 145, 465, 973

myelosuppression 1717

nasopharyngeal carcinoma 530, 1207

national screening programme 174

NCI-60 panel 350

neoadjuvant chemoradiation 924

neoadjuvant chemotherapy 244, 1529

neoadjuvant therapy 1248

neoplasm metastasis 1225

nested case–control study 526

neuroblastoma 55, 342, 1481

neuron 658

NF-*κ*B 1555, 1620

NFAT 1448

NGR-hTNF 219

NIH3T3 829

nitroimidazole 1860

NK cells 1758

non-invasive 1860

non-small cell lung cancer 225, 1537, 1543

North Africa 1207

novel therapies 55

NSAIDs 483

nuclear factor-κB 106

nude mice 1114

obesity 534

occupational activity 1932

oesophageal cancer 855, 1585, 1641

oesophageal squamous cell carcinoma (OSCC) 1298

oestrogen receptor 160, 875

old age 1329

oligonucleotide microarray 1782

oncocytic tumours 132

oncogene 691, 1481

oncogenic mutation 813

opportunistic screening 1925

optical fibre 2015

oral cancer 1194, 1580

oral squamous cell carcinoma 684

oral therapy 232

orthotopic gastric cancer model 1100

osteoblastoma 1909

osteosarcoma 55, 1425

outcome prediction 1074

ovarian cancer 149, 452, 498, 1066, 1107, 1321, 1433, 1461, 1513, 1699, 2023

ovarian carcinogenesis 1107

ovarian neoplasm 504

OVCAR3 269

overdetection 1833

oxaliplatin 357, 1290, 1658, 1846

oxidative stress 452, 1740

*π* class glutathione-S-transferase 1740

p21 691, 816

p21WAF1/CIP1 1433

p53 149, 269, 441, 691, 774, 816, 1606

p53 polymorphism 350

p63 1606

p73 1606

paclitaxel 1059

paclitaxel resistance 1900

paediatrics 518, 722

pancreatic cancer 91, 215, 457, 637, 908, 1145, 1155, 1658, 1709, 1792, 1853

parametric models 902

PARP 256

PC-3 cells 940

PD153035 782

PD98059 782

PDGFA 973

PDGFRA 973

PDT 658

perceived needs 759

perceptions 1502

performance 1074

PGC-1*α* 1253

phage display 645

phase I 1044, 1233, 1241

phase II 615, 1543

phase III 908

phosphatase and tensin homologue 1740

phosphorylation inhibitor 1100

photodynamic therapy (PDT) 1565, 2015

physical activity 1932

phytoestrogens 185

PI3-kinase 145

pickled vegetable 1641

PIK3CA 465

pineal 1613

pituitary adenomas 303

platelet-derived growth factor 1995

platinum 1549

platinum resistance 1321

polymorphisms 256, 1019, 1456, 1817

pomalidomide 803

poorly differentiated thyroid carcinoma 1782

population based studies 1091

post-menopausal 875

PR1A3 1758

pre-clinical 957

prediction of outcome 1282

predictive factors 998

predictive models 1066

predictive signature 1357

preferences 387

pregnancy 1213

pregnancy-associated plasma protein A 278

prevalence 202, 541

primary care 568

prognosis 504, 765, 840, 902, 1005, 1011, 1074, 1175, 1382, 1469, 1529, 1664, 1869, 1957

prognostic 7, 166

prognostic and predictive markers 1824

prognostic biomarker 1137

prognostic factor 287

prognostic marker 457

prognostic value 673

progression 749

proliferation 166, 822, 1005

prospective cohort study 849

prospective trial 1066

prostaglandin 483

prostate adenocarcinoma 1410

prostate cancer 19, 185, 263, 522, 390, 843, 935, 940, 951, 1137, 1225, 1233, 1248, 1620, 1731, 1740, 1833, 1839, 1891, 1932, 1949, 2038, 2043

prostatectomy 1491

proteome 295

proteomics 492, 1145, 1175

PSA 390

PTLD 1019

pyrimidine base damage 452

pyrosequencing 124

QALY 882

quantitative immunohistochemistry 48

quantitative reverse transcription polymerase chain reaction (RT–PCR) 673, 1298

quantum dots 71

R0 metastasectomy 1033

radiation 363, 628

radical prostatectomy 1839

radiological staging 1522

radioresistance 816

radiosensitisation 628

radiotherapy 225, 582, 1839

randomised trial 598

Ras 1555

real-time PCR 605

RecA 1683

rectal cancer 116, 924

recurrence risk 1091

recurrence-free survival 504

refractory solid tumours 1241

registries 1269

renal cancer 238

renal cell carcinoma 1175, 1417, 1876, 2005

replicative stress 363

replicator cell dynamics 1130

residual tissue 1505

resistance 1155, 1357

rhabdomyosarcoma 774, 2030

RhoA 1374

risk 160, 537, 1213, 1456

RNA expression profile 1909

robotic 1491

S-1 225, 1972

S100A6 1145

sagopilone 1241

saliva 1194

sarcoma-initiating cells 2030

satellite glial cell 658

scirrhous-type gastric carcinoma 1365

screening 250, 843, 871, 882, 1269, 1274, 1833

second primary malignancy 935

second primary tumours 1091

second-line chemotherapy 1658

secular trend 174

securin 1005

SEER program 855

selection of extreme phenotypes 1876

self-sampling 871

senescence 1798

sequential therapy 1972

serum 295

serum amyloid A 335

serum bioactivity 160

sexual behaviour 1502

side effects 1502

side population 1425

signal transducer and activator of transcription-3 967

signal transduction 1731

similar expression to FGF (Sef) 1891

Sindbis virus 684

single nuclear polymorphisms 1957

siRNA 957, 1374, 1410

SKOV3 269

small interfering RNA (siRNA) 1798

small molecule inhibitors 774

small-molecule fluorophore 71

Snail 1769

SNP arrays 357, 722

SNPs 1957

socioeconomic factors 897, 1939

somatic evolution of cancer 1130

Sonablate500 19

spatial and temporal distribution 1939

spatiotemporal controlled delivery 1683

specialist care 568

splicing 1183

squamous-cell carcinoma 1, 855, 967

Src 38, 263, 312, 1699

stable shRNAi model 80

stage at diagnosis 215

stage IV endometrial cancer 244

standard gamble 387

standardised mortality ratio 198

stem cell ageing 363

steroid receptor co-activator 1253

STGs 1202

stomach neoplasms 526

stress response 1692

stroma 765

suicide 198

sun bed use 537

sun habits 537

sunitinib 238, 1543, 1876

supradiaphragmatic 582

surgery 91, 395

surveillance 1671

survival 7, 124, 215, 557, 813, 1338, 1513, 1537, 1671, 1749, 1919, 1961, 1978

survival analysis 1199

survivors 541

survivorship 541

sympathetic nervous system 1481

systematic review 238

T cell lines 983

tag SNPs genes 1461

tamoxifen 598, 875, 1769, 1817, 1824

targeted cancer therapy 1683

tasquinimod 1233

TAX 1497

T-cell 1709

telangiectasiae 403

telomerase 1410

temozolomide 124, 615

teratocarcinoma 64

terminal cancer patients 759

tetraspanins 1402

TF-positive microparticle 2023

TGF-*β* 1433

TGF-*β* inhibitor 1884

thalidomide 803

therapeutic resistance 951

thoracic surgery 897

three dimension 473

thymidylate synthase 116

thymoma 1549

thyroid cancer 132, 1630

tissue factor 666, 2023

TMA 673

TNF 1019

TNFα 1555, 1876

tobacco 1207

TOPK 80

toxicity 357

TP53 1456

TRAIL 1683

transcription factor 1481

transcriptional gene silencing (TGS) 1798

transcriptional regulation 132

transformation 1497

transitional cell urothelial carcinoma 1316

translation 424

transperitoneal spread 244

transrectal 19

trastuzumab 782, 1357, 1676

travel 897

treatment outcome 395

treatment variation 1839

TRIB3 1664

tumour budding 1382

tumour growth 1949

tumour hypoxia 1860

tumour imaging 71

tumour markers 335

tumour microenvironment 605, 1869

tumour progression 1410

tumour response 1537

tumour sensitisation 1978

tumour size 902, 1925

tumour spheroids 1290

tumour stage 1671

tumour stem cells 303

tumour suppressor 699, 1651

tumour targeting 645

tumourigenesis 691

TURBT 98

type 2 diabetes 1199

type I IGF receptor 71

tyrosine kinase 312

tyrosine kinase inhibitor 1543

UBE2C 166

UHRF1 98

UK 541

ulcerative colitis 492

unfolded protein response 1692

uPA 432, 1699

uPAR 992

upper tract transitional cell carcinoma 98

uracil-tegafur 598

urinary excretion 185

urine 1175

usual vulvar intraepithelial neoplasia 27

uterine serous papillary cancer 335

uveal melanoma 312, 813

vascular corrosion casting 64

vascular endothelial cell growth factor receptor-3 1100

vascular endothelial cells 106

vascular endothelial growth factor 1986

vascular targeting 219, 1565

VCAM-1 1365

vegetarian diet 192

VEGF 666, 803, 1183, 1417

VEGF_xxx_b 1183

venous thromboembolism 1813

VHL 1417

vitamin D 537, 916

vulvar squamous cell carcinoma 27

Waldenström's macroglobulinemia 1051

walking 1932

Wnt signalling 209, 1909

Wnt-5a 209

wound healing 1393

Y27632 829

young age 1329

younger adults 561, 1939

